# Inhibition of human cytomegalovirus replication by valaciclovir

**DOI:** 10.1099/jgv.0.002178

**Published:** 2025-11-14

**Authors:** Blair L. Strang

**Affiliations:** 1 Institute of Infection & Immunity, St George’s School of Health and Medical Sciences, City St George’s, University of London, London, UK

**Keywords:** cytomegalovirus, drug, pregnancy, valaciclovir

## Abstract

Vertical transmission of human cytomegalovirus (HCMV) during pregnancy is a major cause of congenital disease. In the absence of robust vaccination strategies, antiviral drug regimens are being developed to inhibit the vertical transmission of the virus. Recent clinical data have indicated that valaciclovir, an orally bioavailable form of aciclovir, was effective at limiting vertical transmission of HCMV when administered during pregnancy. However, there is no widely cited *in vitro* analysis of valaciclovir’s antiviral effect against HCMV, and it is possible, like aciclovir, that valaciclovir has poor anti-HCMV activity. The antiviral effects of aciclovir and valaciclovir against HCMV were compared to the widely used anti-HCMV drug ganciclovir. Compared to ganciclovir, the anti-HCMV effects of either aciclovir or valaciclovir were poor, and robust anti-HCMV activity in all cell lines tested (adult fibroblast, foetal fibroblast or trophoblast cells) was only observed at high drug concentrations. All drugs had no obvious effects on the viability of uninfected cells. Overall, valaciclovir had poor anti-HCMV activity, and its anti-HCMV efficacy upon administration during pregnancy may rely on a combination of factors. These data argue for the continued development of valaciclovir and anti-HCMV compounds to inhibit vertical virus transmission.

## Introduction

Human cytomegalovirus (human herpesvirus 5) infection remains a major cause of human morbidity and mortality, with profound impacts on maternal, foetal and child health [1]. Presently, human cytomegalovirus (HCMV) infection is the most prominent congenital infection in the UK and elsewhere, resulting from infection via various routes, including vertical transmission of virus across the placenta *in utero* [2]. Congenital HCMV infection results in a wide range of long-term health issues, such as deafness, blindness and cerebral palsy [1]. The nature of these conditions results in notable social issues and high economic costs associated with long-term care [1].

Various vaccine strategies to control HCMV infection are under development and are being tested in adults [3]. Many are promising, and even limited control of HCMV within the adult population would have beneficial long-term effects on human health [3,4]. However, testing of these strategies during pregnancy or early life is currently very limited, and open questions remain around what strategies should be used to prevent acquisition of HCMV during pregnancy, how to promote vertical transfer of immunity and how to stimulate immunity during early life.

Currently, control of HCMV in all settings is dependent upon the use of antiviral drugs [3,5, 6]. The nucleoside analogue ganciclovir (GCV) has been widely used. Here, the GCV pro-drug is taken up by cells and converted by a combination of both viral (UL97) and cellular kinase enzymes from pro-drug to GCV-monophosphate to GCV-diphosphate to GCV-triphosphate [7,8]. The tri-phosphate nucleotide analogue form of GCV is incorporated into HCMV DNA by the HCMV DNA polymerase, which prevents extension of viral DNA synthesis via an unusual DNA polymerase ‘idling’ process [9]. Because of the need for the viral kinase UL97 protein in the conversion of pro-drug to drug, GCV action is restricted to HCMV-infected cells and has little or no effect on uninfected cell viability *in vitro* [10]. However, *in vivo* administration of GCV to adults commonly has wide-ranging disadvantageous effects, including toxicity and neutropenia. Thus, GCV use must be closely monitored. Additionally, it is widely acknowledged that GCV has a teratogenic effect in animal models [6]. Therefore, GCV is not licensed for use during pregnancy or during early life. However, application of GCV in early life has shown some promise in combating effects of congenital HCMV infection (deafness), and, as yet, no robust data indicate adverse health effects later in life after exposure to GCV in early life [1].

Long-term meta-analysis of HCMV clinical therapeutic intervention has consistently shown that nucleoside analogues such as aciclovir (ACV), and its orally bioavailable form valaciclovir (VACV), have anti-HCMV effects in adults (for example, transplant recipients) that in some settings are comparable to GCV [11,12], although ACV or VACV may have the strongest anti-HCMV effects in the aforementioned settings when used in prophylaxis. ACV and VACV are most commonly used to inhibit replication of other herpesviruses, such as herpes simplex virus (HSV) and varicella-zoster virus (VZV). ACV terminates HCMV, HSV or VZV DNA synthesis, likely using a mechanism distinct from GCV termination of HCMV DNA synthesis [9,13, 14]. However, ACV and VACV are not widely used against HCMV, as it has long been observed that ACV has very poor anti-HCMV effects *in vitro*, with GCV having approximately tenfold greater anti-HCMV effects than ACV [15,16]. This is most likely due to the very poor efficacy of HCMV kinase UL97 catalysing the conversion of ACV pro-drug (nucleoside analogue) to drug (nucleotide analogue) [8].

Regardless, recently, there has been considerable interest in the use of VACV during pregnancy to inhibit vertical HCMV transmission due to its favourable safety profile in humans [17,23], including a recent clinical trial that has demonstrated notable inhibition of HCMV foetal infection when high doses of VACV are administered during pregnancy [24]. The apparent efficacy of VACV in this setting may reflect factors such as the high dose of drug used [17,24]. Alternatively, VACV may have as yet unrecognized efficient anti-viral activity against HCMV in settings related to the vertical transmission.

As mentioned above, ACV has been widely studied in both HSV and HCMV biology. However, there is no widely cited *in vitro* data on the anti-HCMV or anti-HSV effects of VACV, including analysis of VACV activity in settings related to vertical transmission. It is possible these studies have been performed previously and presented either internally in an industrial setting, at previous professional meetings or in journals that are no longer available [25]. The effectiveness of valaciclovir against HCMV *in vitro* could not be established using any artificial intelligence tools, such as ChatGPT (BLS, data not shown). Therefore, analysis of VACV activity *in vitro* against HCMV was investigated, including comparison of VACV to GCV or ACV and testing of these drugs in cell lines relevant to vertical transmission of virus. Ultimately, the anti-HCMV activity of VACV in all settings was poor, implying that antiviral effects of VACV in utero may rely on several factors and future development of VACV and other drugs in the setting of vertical transmission is required.

## Methods

### Drugs

Ganciclovir, aciclovir and valaciclovir were purchased from Insight Biotechnology, SIGMA and Cambridge Biosciences, respectively. All compounds were resuspended in DMSO.

### Cells

Adult human foreskin fibroblast (HFF) cells (clone Hs27) were obtained from American Type Culture Collection, no. CRL-1634 (ATCC, Manassas, VA). Vero cells were a gift from Donald Coen (Harvard). Foetal human foreskin fibroblast cells immortalized with TERT (HFFF-TERT, referred to as HFFF in the text) [26] were a gift from Richard Stanton (Cardiff). Trophoblast cell line SGHPL-4 [27] was a gift from Guy Whitley (City St Georges, London) and was used up to passage 16 [27]. HFF and HFFF cells were maintained in Dulbecco’s Modified Eagle’s Medium (DMEM) (Gibco) containing 10% (v/v) FBS (Gibco) and SGHPL-4 cells were maintained in Rosslyn Park Memorial Institute media (Gibco) containing 10% (v/v) FBS (Gibco).

### Determination of *in vivo* valaciclovir concentration

Variations in dosing have been reported, including 2,000 mg/6 h or 4,000 mg/12 h [18,24]. It has been reported that 2,000 mg/6 h was well tolerated by patients, without toxicity [18]. ChatGTP3 was used to survey available literature, including package inserts of Food and Drug Administration (FDA)-approved drugs, to find the plasma concentration [C_max_ (µg ml^−1^)] of valaciclovir from a 2,000 mg dose. Micromolar concentration of valaciclovir from a 2,000 mg dose was calculated using ChatGPT3 as *C*_max_ × 1,000/molecular weight. Based on a range of the most commonly used *C*_max_ values found by ChatGTP3, the plasma concentration of valaciclovir was calculated as 35–40 µM.

### Herpes simplex virus and human cytomegalovirus

HSV-1 strain 17+ was a gift from Stacey Efstathiou (NIBSC). Virus stocks were generated by low multiplicity infections of Vero cells. HCMV strain Merlin was isolated from a congenital HCMV infection [28,29]. HCMV strain Merlin (R1111) was generated from a bacmid containing deletions in ORFs encoding RL13 and UL128 to allow release of cell-free virus [30]. HCMV stocks were produced in HFF cells. Viral titres of stocks were determined by serial dilution of viral supernatant onto Vero (HSV) or HFF (HCMV) monolayers, which were then covered in DMEM containing 5% (v/v) FBS and 0.6% (w/v) methylcellulose. After incubation for 3 (HSV) or 14 (HCMV) days, cells were fixed with 100% methanol and stained with crystal violet to count plaques in the infected cell monolayers. Titre was expressed as p.f.u. per millilitre.

### Virus yield assays

HFF or HFFF cells were plated at 5×10^4^ cells per well in 24-well plates. After overnight incubation to allow cell attachment, cells were either infected with 5×10^4^ p.f.u. [multiplicity of infection (MOI) of 1] of HCMV or HSV. After virus adsorption for 1 h at 37 °C, cells were washed and incubated with 0.5 ml of media in the presence or absence of drug or DMSO throughout virus replication. Infected cells were incubated for 48 (HSV) or 96 (HCMV) hours at 37 °C before the supernatant was removed from cells for analysis of virus titre by plaque counting, as described in the previous section. Infection of SGHPL-4 cells was carried out as above, except after attachment, cells were maintained in media containing 0.5% (v/v) FBS for 24 h before infection at MOI 1 and incubated in media containing 0.5% (v/v) FBS throughout HCMV replication [27]. EC50 values were calculated using GraphPad PRISM using the non-linear fit of the curve tool (variable slope).

### MTT assays for cell viability

HFF or HFFF cells were seeded at high (1×10^4^ cells per well) or low (1×10^3^ cells per well) numbers cells per well into 96-well plates. High numbers of cells (5×10^3^ cells per well) were used to assess cell viability, whereas low numbers of cells (5×10^2^ cells per well) were used to assess both cell viability and cell proliferation. After overnight incubation to allow cell attachment, cells were treated for 96 h with DMSO or drugs as indicated in the figure legend. 3-(4,5-Dimethylthiazol-2-yl)−2,5-diphenyltetrazolium bromide (MTT) assays [CyQuant (Thermo Fisher)] were carried out on cells in the wells of 96-well plates according to the manufacturer’s instructions. The ability of cellular NAD(P)H-dependent cellular oxidoreductase enzymes to reduce the tetrazolium dye MTT to formazan was measured in a colourimetric assay, read on a FLUOstar Omega Microplate Reader. Analysis of SGHPL-4 cells was carried out as above, except after attachment, cells were maintained in media containing 0.5% (v/v) FBS for 24 h [27] before treatment with either DMSO or drugs. Treated SGHPL-4 cells were incubated in media containing 0.5% (v/v) FBS throughout the assay.

## Results

### Inhibition of herpes simplex virus and human cytomegalovirus replication by GCV, ACV and VACV

**Fig. 1.**
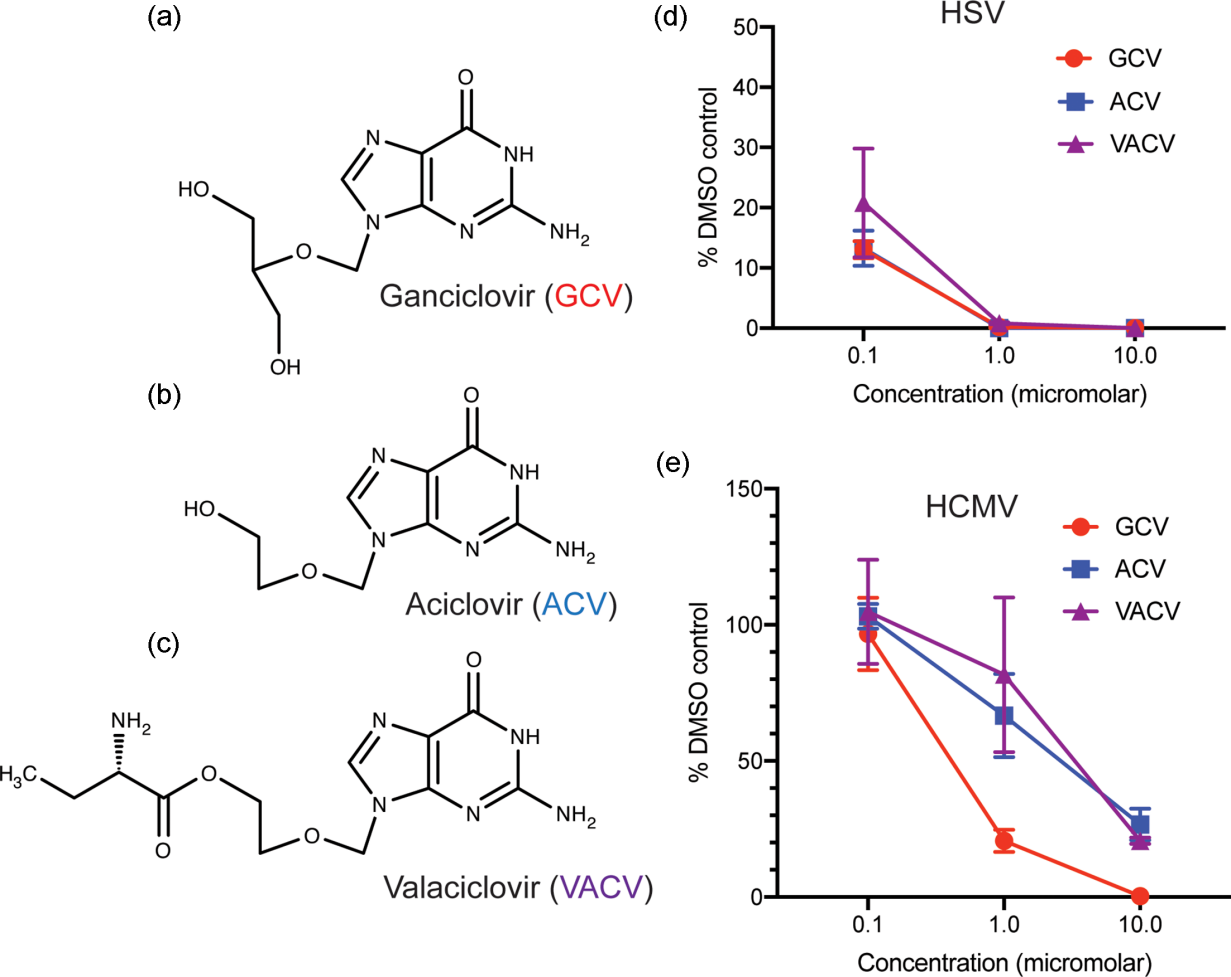
Drugs used in this study and herpesvirus replication in the presence of drugs. The structures of (**a**) ganciclovir, (**b**) aciclovir and (**c**) valaciclovir in their pro-drug forms before conversion to nucleotide triphosphates in infected cells. (**d and e**) In virus yield assays, HFF cells were infected at MOI 1 with either HSV or HCMV in the presence of drug (concentrations indicated in the figures) or the corresponding volume of DMSO. Virus production at 48 (HSV) or 96 (HCMV) hours post-infection is shown as the percentage of infectious virus in the presence of drug compared to the appropriate DMSO control. The data points and error bars in each figure represent the mean of three independent experiments and the standard deviation of those experiments, respectively. At some data points, the error bars are too small to be represented on the figure.

To analyse the anti-HCMV activity of GCV, ACV and VACV (Fig. 1a–c, respectively), drugs were tested for their ability to inhibit HSV in adult fibroblast cells. As expected from long-standing observations [16], GCV and ACV had robust anti-HSV activity, with 50% effective dose (EC50) values well below micromolar concentrations (Fig. 1d). VACV displayed similar anti-HSV activity (Fig. 1d). Consistent with previous reports [8,16], in adult fibroblast cells, GCV had efficient antiviral effect against HCMV, displaying a sub-micromolar EC50 value (EC50=0.5 µM), and the anti-HCMV activity of ACV was poor, with an EC50 value in the micromolar range (EC50=2.7 µM) (Fig. 1e). Like ACV, VACV had poor anti-HCMV activity, with an EC50 value in the micromolar range (EC50=3.4 µM) (Fig. 1e).

### Anti-HCMV effects of VACV in cell lines relevant to vertical transmission

**Fig. 2.**
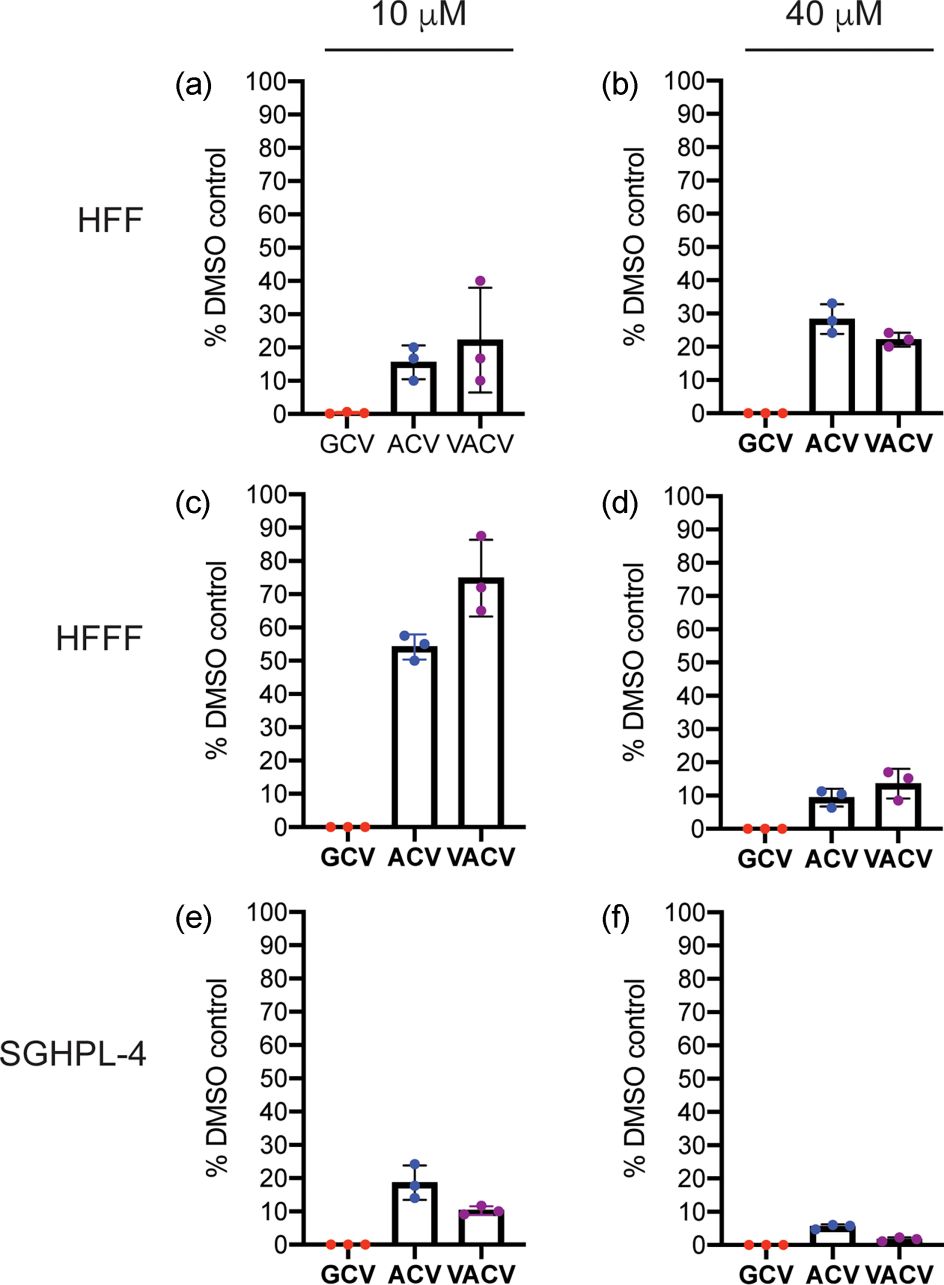
HCMV replication in cell lines. In virus yield assays, each cell line [HFF (a and b), HFFF (c and d) and SGHPL-4 (**e and f**)] was infected at MOI 1 with HCMV in the presence of drug (concentrations indicated in the figures) or the corresponding volume of DMSO. Virus production at 96 h post-infection is shown as the percentage of infectious virus in the presence of drug compared to the appropriate DMSO control. The columns and error bars in each figure represent the mean of three independent experiments and the standard deviation of those experiments, respectively. Data from individual experiments are shown as coloured data points. At some data points, the error bars are too small to be represented on the figure.

Anti-HCMV effects of GCV, ACV and VACV were then tested in both adult (HFF) and foetal (HFFF) fibroblasts, plus a trophoblast cell line (SGHPL-4). SGHPL-4 cells have many features akin to primary trophoblast cells, and our laboratory has previously demonstrated that these cells support HCMV replication [27] (Fig. 2a, c and e, respectively). All drugs inhibited HCMV replication in each cell line. As in Fig. 1, ACV and VACV had less anti-HCMV effects compared to GCV. However, the anti-HCMV activity of both ACV and VACV in HFFF cells was less than in HFF and SGHPL-4 cells. Therefore, it was possible that poor anti-HCMV activity of VACV in foetal fibroblasts may have limited the anti-HCMV activity of VACV *in vivo*. Using a higher concentration of drugs, similar to the concentration of VACV found *in vivo* [18,24] (see the ‘Methods’ section), all drugs robustly inhibited HCMV replication in all cell lines (Fig. 2b, d and f). Therefore, VACV was an inhibitor of HCMV in all cell lines. However, a high concentration of VACV may be required to be most effective in all settings.

### Anti-HCMV activity of VACV was not associated with the lack of cell viability

**Fig. 3.**
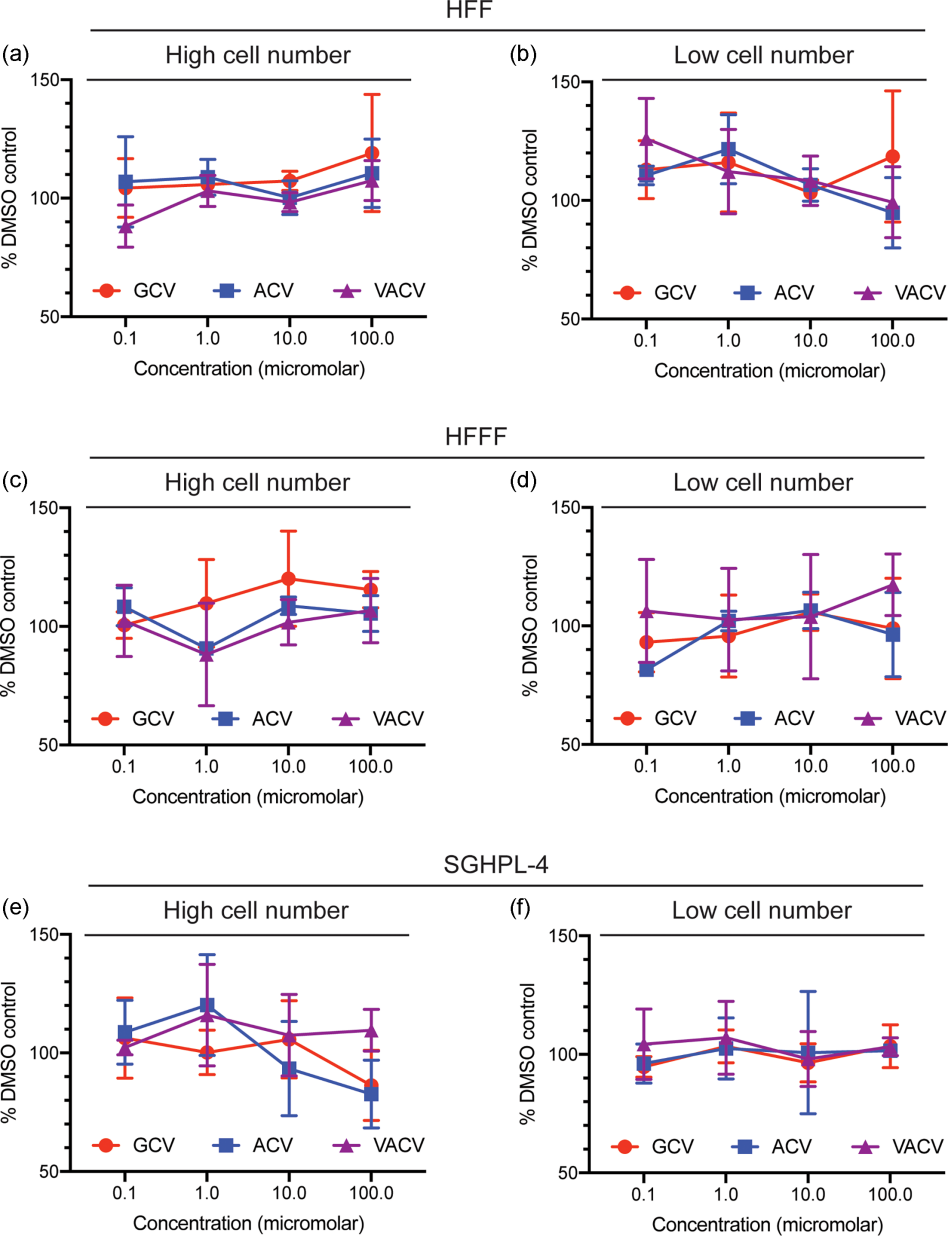
Cell viability and cell division in the presence of drugs. (**a, c and e**) A high or (**b, d and f**) low concentration of each cell line [HFF (a and b), HFFF (c and d) and SGHPL-4 (**e and f**)] was treated for 96 h with concentrations of drug indicated in the figures, or the corresponding volume of DMSO, and then examined using an MTT assay. In each figure, the data point and error bars in each panel represent the mean of three independent experiments and the standard deviation of those experiments, respectively. At some data points, the error bars are too small to be represented on the figure.

The antiviral effects seen above may have been due to defects in cell viability in the presence of drugs, not their antiviral effect. Therefore, we assayed cell viability of uninfected HFF, HFFF and SGHPL-4 cells in the presence of each drug (Fig. 3). High cell numbers (Fig. 3a, c and e) were used to directly assay cell viability, and low cell numbers (Fig. 3b, d and f) were used to assay both cell viability and cell division. Overall, there was no obvious concentration-dependent effect on any cell line by any drug. Therefore, lack of cell viability did not obviously contribute to the lack of virus replication observed in our experiments.

## Discussion

Overall, VACV had poor anti-HCMV activity. It remains unknown why VACV had poor anti-HCMV effects. This may reflect several factors. It is possible that, like ACV [8], the HCMV kinase protein UL97 was ineffective in promoting conversion of the VACV pro-drug form of the compound. Additionally, the cellular enzymes required to promote the conversion of ACV or VACV pro-drug in HCMV-infected cells remain unknown, and it is possible that poor expression of such proteins in HCMV-infected cells contributed to the poor anti-HCMV effects of ACV or VACV. It has also been previously highlighted [8] that the tri-phosphate form of ACV, which is identical to the tri-phosphate form of VACV, has a short intracellular half-life (1–2 h), compared to GCV (15–25 h), which likely contributed to the differences in anti-HCMV effects of these drugs.

Additionally, the poor anti-HCMV effects observed here could reflect inefficient interaction of ACV or VACV with the HCMV DNA polymerase. It was possible that the HCMV DNA polymerase poorly utilized the ACV or VACV tri-phosphate drugs or that once incorporated into HCMV DNA they were ineffective at inhibiting DNA synthesis. Moreover, it remains unknown if ACV or VACV incorporated in HCMV DNA directly terminates HCMV DNA synthesis or, like GCV [9], HCMV DNA polymerase ‘idling’ results in inhibition of HCMV DNA synthesis.

Interestingly, the magnitude of GCV and ACV inhibition of HCMV and HSV varies across different studies [8,15, 16]. For example, approximately fivefold difference between GCV and ACV inhibition of HCMV was found here, whereas a previous study reports approximately tenfold difference between GCV and ACV inhibition of HCMV [16]. Additionally, differences in reported values of GCV inhibition of HCMV can vary greatly, between 0.43 and 7 µM [31]. It is possible these variations arise from dissimilarities in experimental methodology between studies; for example, differences in solutions that drugs are resuspended in, differences in assay methods (virus yield assays versus plaque reduction assays), differences in cell lines used and differences in virus strain used. Moving forward, it may be advisable to develop a standard methodology for testing of anti-HCMV drugs *in vitro*, which would allow more accurate comparisons of drugs’ activity. Additionally, it must be acknowledged that a limitation of this study is the use of the trophoblast cell line SGHPL-4, as they do not recapitulate all the properties of primary trophoblasts and, therefore, are not an ideal trophoblast model system. To better understand anti-HCMV drug efficacy in the future, it will be important to work toward developing new models of trophoblast infection, including the use of primary cells.

Given the poor anti-HCMV efficacy of VACV observed here, it is possible that the protective effects of VACV against HCMV infection *in utero* [24] result from both the anti-HCMV effects of VACV at high concentrations and other factors, which have additive effects. For example, it has been demonstrated that high-dose VACV accumulates *in vivo* during pregnancy [23], which could contribute to anti-HCMV effects. Currently, analysis of this area is hampered by a lack of models for HCMV replication *in vivo* and a lack of primary trophoblast cell cultures that support HCMV infection.

To improve upon the clinical effects of VACV during pregnancy, the use of higher doses of VACV could be explored. As demonstrated here, VACV had no obvious effect on cell viability at high micromolar concentrations. However, it has been observed that high VACV dosing regimens (2 mg/6 h vs. 4 mg/12 h) promote renal failure *in vivo* [18]. Therefore, increasing doses of VACV may not be helpful. Strategies to increase the antiviral effect of VACV and, therefore, use VACV at lower doses would be useful. However, these strategies would likely involve depressing the cellular pool of nucleotides to ensure VACV is always in excess of cellular nucleotides, which is immunosuppressive and could not be used during pregnancy [6]. Many other nucleotide analogues have yet to be tested for anti-HCMV effects. However, as discussed here, these compounds may have good *in vitro* characteristics, but can be problematic when used *in vivo*. Rather, it may be necessary to move away from the use of nucleoside analogues. Direct-acting drugs with proven anti-HCMV activity, such as maribavir and letermovir [3,5, 6], may prove useful in pregnancy and early life but have yet to be robustly tested in these settings. Alternatively, it may be useful to explore the use of host-directed therapy. For example, artemisinin-like compounds have robust anti-HCMV activity [6], and there are few drawbacks to their use during pregnancy.

## Abbreviations

ACV: aciclovir
DMEM: Dulbecco’s Modified Eagle’s Medium
EC_50_: 50% effective dose
GCV: ganciclovir
HCMV: human cytomegalovirus
HFF: human foreskin fibroblast
HSV: herpes simplex virus
MOI: multiplicity of infection
MTT: 3-(4,5-dimethylthiazol-2-yl)−2,5-diphenyltetrazolium bromide
VACV: valaciclovir
VZV: varicella-zoster virus
